# Perioperative care to patients living with HIV by theatre nurses at a South African tertiary hospital

**DOI:** 10.12688/f1000research.125582.2

**Published:** 2024-02-16

**Authors:** Rudzani Ifodia Ngaledzani, Avhatakali Allga Ndou-Mammbona, Azwihangwisi Helen Mavhandu-Mudzusi

**Affiliations:** 1 Department of Health Studies, College of Human Sciences, University of South Africa, Pretoria, 0003, South Africa

**Keywords:** Experiences, interpretative phenomenological analysis, perioperative, tertiary hospital, theatre nurses

## Abstract

**Background:**

Human Immunodeficiency Virus (HIV) is one of the leading health challenges worldwide that influences the provision of quality patient care. Stigma and discrimination around this condition and the health care needs affect the health care provision. This study aimed to gain an in-depth understanding of theatre nurses’ experiences in providing perioperative care to patients living with HIV at a tertiary hospital in South Africa.

**Methods:**

This study was conducted at a tertiary hospital in Tshwane district, South Africa. The study was underpinned by an interpretative phenomenological analysis design. Data were collected from 10 criterion purposively selected theatre nurses using in-depth individual interviews. Data were thematically analyzed and guided by an interpretive phenomenological analysis framework for data analysis.

**Results:**

The study revealed that theatre nurses work in an under-resourced environment. The resources highlighted are human, materials, including personal protective equipment and life support. The situation negatively affects the perioperative care of HIV-positive patients, who are always prone to perioperative complications, such as profuse bleeding, and even death. The situation increases the occupational risk to the nurses.

**Conclusions:**

The researchers proposed several recommendations targeted at improving the resources needed by theatre nurses when caring for people living with HIV perioperatively at South African tertiary hospitals. Findings will add to the body of knowledge of the Nursing profession about offering perioperative care to persons living with HIV.

## Introduction

The rate of Human Immunodeficiency Virus (HIV) infections has increased globally since its discovery in 1980. ^1^ According to Joint United Programme on HIV/AIDS (UNAIDS) ^2^ report, people living with HIV were at 37.2 million globally in 2021. Sub-Saharan Africa takes the lead when it comes to the number of people living with HIV at 71%, with South Africa contributing to the high number of people living with HIV at 8.2 million in 2021 with a prevalence rate of 13.7% in 2021. ^2^ There are several strategies to prevent and reduce the incidence and spread of HIV. The strategies include free condoms supply, Antiretroviral Treatment (ART) rollout, Prevention of Mother to Child Transmission and the UNAIDS 90.90.90. targets, which have ended in 2020, all introduce to curb and manage HIV/AIDS infections. ^3^^,^^4^ The 90.90.90 program aimed to achieve the following: 90% of citizens know their HIV status, 90% of those to be on ART and 90% to have their viral load suppressed by the year 2020 globally. ^3^ South Africa has not achieved the above target due to people’s reluctance to take ART as they are afraid of being stigmatized by health care workers. ^5^^,^^6^

The non-achievement of 90% of adherence in 2020, may still pose a challenge to the achievement of 95-95-95 targets. These revised targets aim at ensuring that at least 95% of all HIV-positive people know their status. Furthermore, 95% of the people who knows their status are put on antiretroviral therapy (ART), and 95% of those on ART are viral suppressed by the year 2030. ^7^

People living with HIV shared different views regarding the attitudes and behaviors displayed by health care workers routine reviews and treatment collection. The above attitudes and behavior are related to wearing double or triple gloves, and extra protective gowns when performing surgical/medical procedures, and refusal to perform some medical/surgical procedures on people living with HIV. ^8^ Factors leading to these stigmatizing behaviors and attitudes among health care workers are complex as they emanate from individual, hospital and politics. ^9^^,^^10^

Religious beliefs, the age, and gender of the health care workers are the main individual causes leading to stigmatization of people living with HIV. Bureaucracies and hospital policies also have an influence on the management of people living with HIV. ^10^ Hospital policies and bureaucracies are the leading factors in stigmatization. Some authors, ^10^ reported that health care workers turned away patients living with HIV citing that the hospital concerned is not designated for people living with HIV and AIDS. A lack of availability of Post Exposure Prophylaxis (PEP) in the hospitals make some health care workers reluctant to render care to patients living with HIV. ^11^

Reluctancy to care for patients living with HIV and lack of knowledge about PEP by some of the health care workers also contribute rendering of substandard care as some patients are turned away even in hospitals where PEP is available. ^12^ Some healthcare workers’ attitudes towards patients living with HIV are based on a limited understanding of the risk related to their specialization and cultural setting. ^10^^,^^13^^,^^14^ Therefore, the aim of this study was to gain an in-depth understanding of the provision of perioperative care by theatre nurses to patients living with HIV at a tertiary hospital in South Africa.

## Methods

The qualitative research approach was used as the researchers wanted to have an in-depth empirical understanding of a phenomenon in its natural setting. ^15^ The researchers used this approach because they wanted to understand the provision of perioperative care rendered to patients living with HIV by theatre nurses to patients living with HIV at a tertiary hospital in South Africa. Interpretative phenomenological analysis design was used in the study. Interpretative phenomenological analysis is a research design used to explore in detail how participants are making sense of their personal and social world. ^15^^–^^17^

### Ethical considerations

The Department of Health Studies, REC-012714-039 (NHERC), and the Research Ethics Committee of the University of South Africa granted the researchers ethical clearance for the study (Ethics Clearance Number HSHDC/987/2020) on 5 June 2020. Permission was also obtained from the Gauteng Department of Health and, the Chief Executive Officer of Kalafong provincial tertiary hospital where interviews were conducted. The researchers also obtained written voluntary informed consent from all participants in the study. Throughout the study, ethical principles were observed by using pseudonyms to ensure confidentiality, respect, privacy, and protection of the participant, researchers, and institutions. The researchers only documented information for which permission was given by each participant.

### Setting

This study was conducted in one of the tertiary hospitals in the Tshwane district, which has 12 operating theatres with different areas of specialization. There is one Endoscopy unit, which is situated in the Out Patients Department (OPD), two Obstetrics theatres that are allocated in the Maternity section, Future Laparoscopy theatre, two Orthopedic theatres, Clean emergency theatres, Urology theatre, Ear, Nose and Throat (ENT) theatre, Ophthalmology theatre, Septic emergency theatre, Neurology theatre, Pediatric theatre, and a General surgery theatre. The reason for choosing the tertiary hospital is because of being situated in a township, and serving mostly patients who are from the local townships (semi urban places) who are either brought directly from their homes as an emergency or referred from either districts or regional hospitals because of complications from districts hospitals all over South Africa. These situations make the hospital theatres to be always busy.

### Population

The population of this study was all the theatre nurses of a tertiary hospital in the Tshwane district who meet the inclusion criteria and voluntarily agreed to participate. To recruit the participants, the first author presented the study to all theatre nurses who were on duty during different shifts. The potential participants were given information leaflets with all the relevant information regarding the study and the cell numbers of the research team. Those who were interested to participate in the study contacted the researcher using WhatsApp messages or using a ‘call back’ request. The researcher made a follow-up call to further discuss the study.

### Sample and sampling methods

The sample size for this study was 10 purposively selected theatre nurses who met the following inclusion criteria: being a theatre nurse; at least two years of experience working in theatre; aged 25 to 65 years of age. Only theatre nurses who agreed and offered voluntary consent participated in the study. The sample size was determined by data saturation. ^18^ Data saturation is the point where no new information is emerging during the data collection process and analysis, which in most the qualitative studies occurs iteratively. ^19^

### Demographic data of participants

This section provides information concerning the demography of the study participants, which are displayed in Table 1. The information included are participants’ age, sex, years of experience, and qualifications. Demographic data are included to assist the reader in understanding the source of data. All participants were females. This is because there are only two male, professional nurses, in the whole theatre complex. Both of them were not accessible during data collection as they were working during the night.

**Table 1.** Demographic data of participants.

Participant’s pseudonym	Age, years	Marital status	Years of experience	Qualifications
Rofunwa	37	M	03	R425
Avhatakali	34	M	13	R425, R212
Azwimpheleli	44	M	20	R425, R212
Mukona	29	S	03	R425
Munyadziwa	55	S	09	R2175, R683, R212, R254
Ntshengedzeni	45	M	08	R683, R254
Murida	50	S	10	R425, R212
Muthelo	56	M	20	R2175, R683, R254, R212
Avheani	50	S	20	R2175, R683, R254, R212
Malindi	33	M	06	R425, R212
**Participants, total number**	**Age, years (total number)**	**Marital status (total number)**	**Experience, years (total number)**	**Qualifications (total number)**
10	20–30 (1) 31–40 (3) 41–50 (2) 51–60 (4)	Married (6) Single (4)	02–05 (2) 6–10 (4) 11–15 (1) 16–20 (3)	Single (1) Multiple (9)

R2175=South African Nursing Council Regulation for a course leading to registration as a Nurse. R683=South African Nursing Council Regulation for a course leading to registration as a General Nurse. R425=South African Nursing Council Regulation for a course leading to registration as Nurse (General, Psychiatry, Community) and Midwifery. R254=South African Nursing Council Regulation for a course for a diploma in midwifery leading to registration as a Midwife. R212=South African Nursing Council Regulation for a course leading to registration as a Nurse Specialist or Midwife Specialist (clinical) and Health Service Manager and Nurse Educator (nonclinical).

### Data collection instrument

To cover the list of questions in an interview guide, the researcher used in-depth semi-structured interviews. The interview guide was written in English as it is the medium of communication that all theatre nurses understand well. Piloting was done by interviewing one theatre nurse who was not part of the study. The pilot interview lasted for only 10 minutes and was audio-recorded. The research team listened to the audio recording and discussed the questions where the corresponding author, who was the study supervisor, analyzed the question asked and the response. She highlighted that some of the probes were closed-ended and leading. The corresponding author also highlighted that probing was inadequate as most of the participants’ responses seemed to be ambiguous or incomplete sentences, but no further questions were asking for further clarification. She further demonstrated how IPA interviews are conducted through interviewing the second author. The first author was then requested to conduct a second interview, which on review was considered appropriately done. A permission was granted to the first author to continue with data collection. The interview guide can be found as *Extended data*. ^36^

### Data collection procedure

Seven participants were interviewed at the hospital premises in the theatre seminar room, two in their homes and one at the restaurant of choice. To ensure privacy in the seminar room, a notice was written on the door stating ‘meeting in progress’ to avoid disturbance. For those who preferred the restaurants, we used private tables where there were minimum disturbances. Each interview was initiated by a general conversation focusing on biographical data such as age, marital status, qualification, and experience in theatre. This was followed by the following question: “ *kindly share with me your experiences of perioperative care to patients living with HIV”.* Pre-set questions were used in case where the response to the initial question did not cover them. Probes and follow-up questions were guided by the participants’ responses. The recording was done using an audio digital smartphone of high quality. Each interview lasted for 30–45 minutes. The interview was transcribed verbatim within 24 hours. The original recordings were transferred to a memory stick and deleted from the phone. To ensure confidentiality, the memory stick is kept under a locked cupboard together with the transcripts. These documents will be destroyed after five years. After participant number seven, data saturation was reached, but the researcher continued to interview three more participants searching if there would be new information given. Some scholars ^20^ believe that data saturation is commonly referred to as a point where collected data or analyzed yields no further new information and further data collection is no longer necessary. Field notes were also taken concurrently with the interviews. All COVID-19 protocols were observed through sitting 1.5 meters away from the participant, sanitizing hands and surfaces, wearing masks, and ensuring proper ventilation. The interviews were done from May 2021 to June 2021.

### Data analysis

The data were manually analyzed iteratively with data collection guided by an IPA framework for data analysis. The framework was considered most suitable as the researchers’ focus was not only to analyze, but also to interpret the findings by focusing on how participants interpret their own experiences. ^21^ The following seven steps were followed:

*Read and re-reading:* In the first step, the researchers repeatedly listens to the recorded interviews, read and re-read the transcripts to have an in-depth understanding. By so doing the researchers were able to correct mistakes that occurred during the verbatim transcribing of participants’ interviews.

*Initial noting:* In this second step the researchers searched for similarities in each participant’s transcripts and noted them.

*Developing emergent themes:* The researchers grouped the notes into categories of themes.

*Searching for connections across emergent themes:* The researchers merged similar categories, to develop super-ordinate themes, themes, and sub-themes. 

*Moving to the next case:* After searching for connections across the emergent themes, the researchers summarized the emergent themes. Rechecking all the themes considerately to find out if there are no new emerging themes.

*Looking for patterns across cases*: The researchers discussed their individual themes and further compressed the themes into smaller themes after checking for patterns in the themes and renamed them.

*Taking interpretations to deeper levels:* This is the seventh and last step; the researchers used current ideas to contextualize the final themes that emerged from the process. Three super-ordinate themes with themes and sub-themes emerged from data analysis: human resources, material resources, and infrastructural resources.

### Measures to ensure trustworthiness

To ensure rigor for the study, Guba and Lincoln’s (1994) framework for ensuring trustworthiness was followed. Guba and Lincoln’s criteria of ensuring trustworthiness included credibility, confirmability, dependability and transferability. ^22^ The researchers used the interview guide and audio recorded the interviews, which were transcribed verbatim to ensure credibility. To ensure transferability, the researcher appropriately described the study design, setting, population, sampling, sample, the study’s key informants, data collection, and analysis process to ensure transferability and dependability. To avoid presenting the individual researcher’s biases or perspectives, all the researchers independently analyzed data, came up with a table of themes, and later compared and discussed their themes until they constructed a single master table of themes to ensure confirmability.

### Reflexive statement

All the researchers are nurses by profession though authors number two and three are now working in the Institution of Higher Education. The first author, who also collected data is a theatre trained nurse who is also a manager of the theatre. Her experience and her position as a manager might have affected how she collected data. However, this was curbed through first piloting data collection and refining how questions should be asked. Moreover, the interviews were audio recorded, transcribed verbatim in preparation of analysis by all researchers who also listened to the audio recordings. 

## Results

The themes that emerged from data analysis are presented in Table 2. This section provides a summary of the results provided based on super-ordinate themes, themes, and sub-themes that emerged from data analysis. Three superordinate themes emerged from data analysis, which included: human resources, material resources, and infrastructural resources.

**Table 2.** Summary of the results.

Superordinate theme	Theme	Sub-theme
**Human resources**	***Nursing Staff***	*Shortage of nurses during all shifts.*
		*Limited knowledge by nurses regarding post-exposure prophylaxis.*
	***Other members of the support team***	*Lack of consultants after hours.*
		*Limited time for rendering preoperative care.*
**Material resources**	***Personal protective equipment***	*Shortage of personal protective equipment.*
		*Discriminatory supply of protective material.*
	***Pharmaceutical resources***	*Availability of pharmaceutical resources to treat complications.*
**Infrastructural resources**	***Space in theatre***	*Inadequate space for nursing complicated patients in theatre.*
		*Limited space in the Intensive Care Unit (ICU) where the complicated patients can be transferred to.*
	***Location of the theatre***	*Proximity of theatre to the blood bank.*

### Human resources

This superordinate theme focuses on challenges experienced by the theatre nurses regarding resources while rendering perioperative care in one of the tertiary hospitals in the Tshwane District. It is composed of the following themes: nursing staff and other members of the support team.

*Nursing staff*

Human resources in terms of nursing staff and consultants were found to be the most challenging when providing care during the perioperative period to a patient living with HIV by theatre nurses. The theme comprises the following subthemes: shortage of nurses during all shifts and limited knowledge regarding post-exposure prophylaxis.

Shortage of nurses during all shifts

Participants mentioned that there is no enough human resource during all shifts. The study also revealed that there is scarcity of theatre nurses who can relieve and give reports in time. The following participants’ excerpts support the above statement:

*“We are not well staffed. Sometimes we are only two and the other person will go off earlier and there is no one to relieve me, especially with Caesarean Sections. I must attend to dire emergencies like fetal distress as I am the only one. There is a gross shortage of staff. I sometimes do not even have time for a break. I am just expected to go on with the operation list.” (Rofunwa)*

*“There is no staff to relieve us. Shortage of personnel is a huge problem in the department. These long procedures that complicate drain me physically and emotionally. There is no one to relieve me. I must go back and face another patient as if the previous case never happens.” (Avhatakali)*

Limited knowledge regarding post-exposure prophylaxis

The challenge related to human resources is not only shortage but also limited knowledge on HIV PEP among nurses. The study indicated that some of the theatre nurses who are supposed to have pharmaceutical knowledge of medicine do not even know the names of the antiretroviral medication given as prophylaxis following exposure to HIV-contaminated body fluids. Supporting the above are the following participants’ quotations:

*“I don’t know the name of the tablets, I have not been in the situation, but what I know is that they are antiretrovirals”. (Mukona)*

*“I don’t remember the name, but it was a combination pill with Nevofir. I do not remember the other drugs”. (Munyadziwai)*

*Other members of the support team*

The lack of other support staff in theatre negatively affects the provision of perioperative care in theatre during emergency procedures. The above theme consists of the following sub-themes: lack of consultants after hours and limited time to render preoperative care.

Lack of consultants after hours

Apart from the shortage of nurses during all shifts, there is always a lack of consultants after hours who are supposed to work during operations. For example, the anesthetist consultant only works during office hours. This poses a challenge for the anesthetist registrars as they are unable to execute certain functions or operate some of the equipment in theatre after hours.

“ *The consumables of the cell saver machine are not readily available, and some of the anesthetist does not know how to use the cell saver machine. It is normally their consultant who operates that machine. If it is after hours and during the night when the consultant is not there, we cannot use it even if consumables are available. And this put us in danger of losing patients, especially if HIV patients start to bleed.” (Azwimpheleli)*

Limited time to render preoperative care

Apart from the above concerns, another challenge related to the human resource is limited time to render preoperative care. It was indicated that consultants and theatre nurses do not have an opportunity to visit the patients a day before the operation to conduct pre-operative care due to workload. This is shown by the following excerpt from one of the participants:

“ *You are supposed to do the preoperative visit, but due to workload, we do not do it. I only meet my patient at the reception area on the day of the operation. This is because we only know where we are supposed to work in the morning.” (Ntshengedzeni)*

### Material resources

The challenge is not only limited to human resources, but also a shortage of material resources. This superordinate theme is composed of the following sub-themes: personal protective equipment (PPE) and pharmaceutical resources.

*Personal protective equipment (PPE)*

Without PPE (gloves, protective apron, google and visors), it is difficult for theatre nurses to render some of the procedures where there is fluid contact, for instance, delivery of a baby (cesarean section) and laparotomy (opening of the abdomen).

*“The department does not care about us. How can one assist during a cesarean section without visors when it is known that there is a possibility of blood splashing? It is prone for one to get infected with HIV in the process of delivering a child”. (Malindi)*

Shortage of PPE

Participants indicated a shortage of PPE such as masks, gloves, google, and visors from the theatre department, even from the hospital central storeroom at the procurement department.

*“Some PPE are readily available like masks, but sometimes we do not have all the sizes of gloves, especially small sizes gloves, because most of the staff members wear from size seven, so the ensure consistent supply of that size. They hardly order smaller sizes making us with tiny hands struggle. If I find size six gloves, I must hide them in my locker so that I don’t struggle in the future when there is no stock. Items such as protective plastic aprons and masks are always available. The goggles are not always there, and we end up purchasing our own goggles. The visors are a serious problem. If you get one, you must finish the whole list with it, and it smells. The surgical masks are easier to get everywhere as it is Covid-19 times.” (Murida)*

Discriminatory supply of protective material

Though there is a shortage of personal protective clothing, there are also discriminatory supply practices between the nurses and doctors. Some participants indicated that certain material resources are distributed with favoritisms between the doctors and the nurses.

*“To be honest, the goggles you struggle to get them. They are not readily available to nursing staff, but the doctors are always issued goggles. The anesthetist will tell you that they are buying the goggles themselves or their seniors are buying for them, which we are not even sure of. For nursing staff, the goggles are there, but we are so many, and they are few. Some use them and put them in their lockers instead of putting them in their designated area. The whole department is issued with goggles, but once they break, a person must wait for their turn when they are issuing for everyone.” (Muthelo)*

*Pharmaceutical resources*

The study indicated that there is always availability of pharmaceutical supplies, for instance, frozen dry plasma, which makes the job easy for theatre nurses when there is an emergency in theatre regarding patients living with HIV as most of them need plasma because of their low hemoglobin level.

Availability of pharmaceutical resources to treat complications

According to the study results, there is a good supply of pharmaceutical products between the theatre and pharmacy departments. There is also good management of these pharmaceutical products as most participants indicated that there is always something to utilize in emergencies and routine lists.

*“We are using frozen dry plasma. It comes as a dry powder that we mix with two hundred milliliters of saline. The frozen plasma is from the blood bank, and this one is from the pharmacy, and it is always available. This is our first line of defense”. (Avheani)*

*“We always have resuscitation fluids at all times like voluven, ringer’s lactate and hemostats like spongostan and surgicell.*” ( *Malindi)*

### Infrastructural resources

Even though pharmaceutical supply is not a problem, there are some challenges regarding infrastructure. The above superordinate theme consists of the following sub-themes: space in theatre and location of the theatre.

*Space in the theatre*

The other problem raised is the limited space in the theatre where patients need to be monitored after operations have been performed before they can be sent to the intensive care unit or the various wards.

*“It is difficult to observe a patient in theatre after surgery due to limited space. The patients are transferred to the wards regardless of their condition. The above is done to avoid crowding in the theatre observation room, which might also lead to cross infections, as we are dealing with patients living with HIV”. (Rofunwa)*

Inadequate space for nursing complicated patients in theatre

The geographical plan of the theatre department of the hospital under study has 13 theatres in total, which are divided into different areas of specialization. There is one endoscopy unit, two obstetrics theatres, two orthopedic theatres, one future laparoscopy theatre that is shared by all disciplines according to days and time of allocation, one Gynecology theatre, one urology theatre, one ENT theatre, one Ophthalmology theatre, one General Surgery theatre, and two Emergency theatres (clean and septic). There is only one recovery room for the whole complex with six recovery cubicles. There is no Boyles Machine in the recovery unit where complicated patients can be nursed. The patient is nursed in the theatre where the operation takes place.

*“The patients are being resuscitated in theatre until they wake up or until the anesthetist finds an intensive care unit bed or high care bed. If there is no bed, the patient is nursed in the theatre department by the whole team.” (Azwimpheleli).*

Limited space in the Intensive Care Unit (ICU) where the complicated patients can be transferred to

Apart from the theatre unit where patients were operated on, there is a challenge of limited space in the ICU where patients must be transferred to post-operation. The results of the study indicated that there are only eight ICU beds in the hospital under investigation, which is a tertiary institution.

*“No, the intensive care unit beds are only eight. If it is full, the patient is nursed in the operating theatre, being ventilated until there is a bed available. Most of the time, we arrange an intensive care bed from other hospitals. If we don’t get a bed, it only means that a nurse from theatre must nurse this patient together with the anesthetist”. (Munyadziwa)*

*Location of the theatre*

Even though the patient got stuck in the theatre due to complications and the unavailability of ICU beds, it was not difficult for staff members to get blood products to the theatre complex.

*“The blood bank is next to our theatre, so there is no problem in getting blood on time. There is no delay when it comes to the much-needed blood plasma in theatre.” (Avheani)*

The proximity of theatre to the blood bank

Results of the study indicated that the proximity of the blood blank is near the main theatre which makes it easy to obtain blood in case of emergency.

*“Yes, we have a blood bank, and fortunately, it is closer to the theatre. If there is no intern doctor to go collect blood, we just send our general worker to go and collect.” (Rofunwa)*

## Discussion

### Human resources

The shortage of human resources, especially professional nurses due to retirement, and absenteeism worsened by contracting the coronavirus is creating many challenges. Most of the time, nurses are allocated in the theatre alone without anyone to relieve them. This creates a challenge because, for patients living with HIV, their procedure takes longer than that of others due to their proneness to complications. In a study conducted in New Zealand ^23^ it was found that there is a tremendous loss of human capital in healthcare facilities. Not just a shortage of nurses but a shortage of experienced nurses. Therefore, the hospitals ought to recognize that their most senior nurses are a valuable resource and that they might extend their careers by offering some of them opportunities to become mentors.

The study also revealed that even though nurses under training are allocated in the theatre department, there is a need to retain highly experienced and highly trained personnel to transfer the knowledge to the students and newly qualified employees. The same sentiment was shared in the study conducted by one scholar, ^24^ that hospitals are using nurses who are supposed to be training to patch their shortage and those nurses are forced to work without supervision, as it was supposed to be.

The study also finds that not only limited nursing resources but also the anesthetist consultants are unavailable after hours. That means that the anesthetist registrar does not conduct certain functions due to difficulties in operating some equipment. The same sentiment was shared by other scholar ^18^ that the lack of anesthesia providers means that more people may die in poor countries during operations or due to postponement of operations, and a smaller number of surgeries will be performed by people with poor or no training in anesthesia.

The results indicated that some professional nurses lack knowledge of PEP. This is due to their nature of work. They do not deal with oral medication in theatre as patients who come to the theatre are starved; they only concentrate on anesthetic agents and intramuscular analgesics. Similar findings were reported in the study conducted in Bhutan, ^25^ among 221 registered nurses working at the hospital regarding knowledge of PEP. Their results revealed that 80% of the participants had poor knowledge regarding PEP.

### Material resources

Protective materials that are continuously available are face masks, especially because of the protocol for the management of the COVID-19 pandemic. With other materials, such as gloves, only people who put on size seven gloves are usually catered for. Those who wear smaller sizes such as size six or six and a half are most of the time not catered for. They are therefore forced to put on oversized gloves, which make them more prone to be pricked. Sometimes they are forced to put on smaller available sizes, which are very uncomfortable. Sharing the same sentiment is a previous study conducted in South Africa, ^13^^,^^26^ which stated that shortage of other protective equipment is making it hard for nurses to provide care without being afraid of contracting HIV in the process.

The type of duties they perform perioperatively makes them prone to be exposed to HIV-contaminated body fluids. The challenge is further worsened because of the lack of protective clothing, such as goggles and visors. This compromises their health especially when the patient is HIV positive as there is a possibility of blood gushing into their eyes and predisposing them to infection. Other scholars ^27^^,^^28^ reported that professional nurses raised their concerns in one of the hospitals in the Tshwane District about the supply of protective clothing that was used as a precautionary measure to prevent the transmission of HIV from patient blood and body fluid.

The study findings also revealed that the shortage of protective materials seems to be discriminatory as, while nurses may not be provided with adequate quality protective equipment, the anesthetists and surgeons are always provided with that material. This means that other members of the health care team are given priority when the PPE is scarce while all team members involved with the patient perioperatively are at risk of contracting the virus. Similar results were shared by other authors, ^29^ highlighting that the staff that is working at National Health System are required to wear different types of PPE depending on their clinical setting according to their level of exposure to infectious conditions such as HIV.

This study also found that, although there is a shortage of protective materials, pharmaceutical products to help resuscitate HIV-positive patients when they are complicated, especially bleeding profusely, which is common among HIV-positive patients are always available. The available pharmaceutical products include Ringer’s lactate solution, Voluven, Cyklokapron, and dry plasma to help with resuscitation when these patients start to bleed excessively. Contrary to the above statement, a study conducted in South Africa, ^30^ revealed that there is always a shortage of pharmaceutical materials, and medicine in public hospitals, which makes it difficult to provide quality care to the patient.

### Infrastructural resources

The findings indicate the shortage of infrastructure that affects the care of HIV-positive patients if they have perioperative complications. They mentioned that the hospital has several theatres, which most of the time will be utilized simultaneously. According to a study conducted in South Africa, ^31^ shortage of infrastructure contributed negatively to the management of people living with HIV. This is because, after operation, people living with HIV require adequate space with all necessary equipment where they could be nursed until they fully recover. Despite so many theatres, the hospital has only one recovery room for the whole complex with six recovery cubicles. There is no Boyles Machine in the recovery unit where complicated patients can be nursed. This means that if a patient has complications, they are nursed in the theatre where the operation takes place until they recover. That means that a nurse who has scrubbed for the patient has to be with a patient living with HIV for a very long time. Sharing the same narratives is a study conducted by another author, ^32^ which found that lack of proper resources contributes to the preventable death or complications of patients.

The other challenge is that it becomes very difficult to get a bed in the ICU for HIV-positive patients postoperatively as there are only eight ICU beds in the hospital, which is a tertiary academic hospital that operates many major operations booked routinely and those major cases on emergency from all over South Africa, and even the neighboring countries. This is in contrast with the recommendations by another author ^33^ stating that health care sites, including hospitals, should be integrated with the broader community to promote accessibility. The finding related to limited ICU beds, making it difficult to get a bed for a complicated HIV patient attest to the findings by a study done in South Africa ^34^ that the last national audit of ICU resources in South Africa indicated that there were 4,179 ICU and high care beds in the private and public sector in South Africa to cater for 57 million population of the total number, 75% of beds (3,533) were in the private sector, and only 25% (1,186) were in the public sector, which alone caters for 84% of the population. Gauteng is allocated 49%, which is 2,312 beds, in both the public and private sectors. The other positive thing is the proximity of the theatre to a blood bank, which makes it easy for one to get blood during an emergency and it also fastens the nursing care of the patients and prevents complications or death. Contrary to the above, as shared in a study conducted in Chhattisgarh, ^35^ the proximity of the blood bank to the theatre is very important as it also reduces delays that can lead to complications and death.

The results did not only show the negative aspects related to infrastructure but also showed the positive aspects. The positive issues are that the blood bank is situated next to the main theatre. Though it is a bit too far from the maternity theatre, it is still a walking distance where an intern doctor or the general theatre worker could walk and collect blood. This has assisted in saving the lives of most HIV-positive patients who have complications during surgery. Another author ^35^ agrees with the above that if the blood bank is nearer, it is good for saving patients’ lives before they have serious complications or die.

### Limitations

The participants were only professional nurses while there are also other categories of nurses who work in theatre. The study was conducted during COVID-19 lockdown, and because of wearing the mask and social distancing, it was difficult to observe some non-verbal cues as the mouth and nose were covered. It was also difficult to maintain eye contact with the participants as some participants put on a face shield. Only female theatre nurses have participated in the study which limit the understanding of gender variation in experiences. This might have affected the observations of some cues which necessitated further probing.

### Recommendations

The hospital needs to provide adequate staffing in the theatre to ensure that theatre nurses are not overstretched as exhaustion leads to poor quality care for patients living with HIV perioperatively. Supply chain or procurement department need to supply enough protective equipment to theatres as most participants indicated a decline in quality perioperative care due to lack of consumables. The hospital should supply theatre staff with adequate quality protective clothing and safety materials such as masks, plastic aprons, and disposable gloves. The government needs to construct a high-standard hospital infrastructure that is adequate and adaptable for the required patients’ services. Moreover, the hospital should come up with the strategies to enhance the healthcare workers’ knowledge regarding HIV post exposure prophylaxis incase they are exposed to body fluids of patients with HIV.

## Conclusions

Results indicated that professional nurses caring for people living with HIV experience a shortage of human resources, material resources, and infrastructural resources. The study indicated that professional nurses in the theatre department fail to provide adequate quality care to people living with HIV perioperatively due to inadequate resources and lack of support. Though the experience related to resources does not only affect Perioperative care of HIV positive patients, but its impact is also worse on this population were majority of them are immune compromised. If the situation remains the same, it may be impossible to reduce HIV related death. Moreover, the institution might end up losing a lot of money due to litigation related to occupational injuries or death of patient following negligence during operation. The government should ensure that, theatre nurses are provided with adequate personal protective clothing, theatres are adequately staffed and equipped with all necessary equipment, so that theatre nurses can provide quality nursing care to people living with HIV perioperatively. 

## Data Availability

The transcripts and audio-recordings for this study are available from the first authors on request ( mmudza@unisa.ac.za or rngaledzani@gmail.com). We could not openly upload data on the website because the transcripts are verbatim. Moreover, when reading those transcripts though the identifiers such as biographical data are removed, people from the hospital where data were conducted could easily link the narratives to a specific participant based on the certain manner in which specific people express themselves. Zenodo: Prof. https://doi.org/10.5281/zenodo.7278297. ^36^ This project contains the following extended data:
- Interview guide 2.docx Data are available under the terms of the Creative Commons Attribution 4.0 International license (CC-BY 4.0).
