# Supplementary material for: Perioperative care to patients living with HIV by theatre nurses at a South African tertiary hospital
Source: F1000Res. 2024 Feb 16;11:1299. Originally published 2022 Nov 14. [Version 2] doi: 10.12688/f1000research.125582.2 (PMC10951563; doi:10.12688/f1000research.125582.2)
Supplement: Peer review history

# Reviewer report: Reviewer response for version 2

**DOI:** 10.5256/f1000research.162660.r247971

**Published:** 2024-03-19

**Authors:** Nnamdi Ndubuka

**Affiliations:** 1 Community Health and Epidemiology, University of Saskatchewan, Saskatoon, Saskatchewan, Canada; 2 Public Health, Northern Inter-Tribal Health Authority Inc., Prince Albert, SK, Canada; 3 School of Public Health, University of Saskatchewan, Saskatoon, Saskatchewan, Canada

I approve the current version.

Is the work clearly and accurately presented and does it cite the current literature?

Partly

If applicable, is the statistical analysis and its interpretation appropriate?

Partly

Are all the source data underlying the results available to ensure full reproducibility?

Yes

Is the study design appropriate and is the work technically sound?

Yes

Are the conclusions drawn adequately supported by the results?

Partly

Are sufficient details of methods and analysis provided to allow replication by others?

Yes

Reviewer Expertise:

Sexually Transmitted and Blood Borne Infections; Social epidemiology and determinants of health; Population health intervention research; Indigenous Health; Clinical quality improvement; Epidemiology

I confirm that I have read this submission and believe that I have an appropriate level of expertise to confirm that it is of an acceptable scientific standard.

# Reviewer report: Reviewer response for version 1

**DOI:** 10.5256/f1000research.137906.r204205

**Published:** 2023-11-29

**Authors:** Nnamdi Ndubuka

**Affiliations:** 1 Community Health and Epidemiology, University of Saskatchewan, Saskatoon, Saskatchewan, Canada; 2 Public Health, Northern Inter-Tribal Health Authority Inc., Prince Albert, SK, Canada; 3 School of Public Health, University of Saskatchewan, Saskatoon, Saskatchewan, Canada

The authors clearly articulated the aim of the study. The study is very essential recognizing the need for South Africa to reach the UNAIDS 90-90-90 targets.

**Research design**

 The use of a qualitative research methodology is appropriate for this study. The authors provided a clear justification for the choice of method. The research design also enabled the authors to explore and describe the experiences of theatre nurses in caring for patients living with HIV. 

**Literature review**

 The authors demonstrated adequate knowledge of the research topic. Literature review process was appropriate and in-line with study objective. However, the literature review section could be strengthened by including the new set of UNAIDS target of 95-95-95 released in December 2020.

**Research setting:**

 It would be reasonable for the authors to provide a rational for choosing the tertiary hospital in Tshwane district. What informed this choice and not other facilities?

**Inclusion criteria:**

 Having 2years of experience working in the theatre was identified as a criteria to participate in the study. The authors need to reword this criteria as those more than two years of experience were also included. Suggested wording: "at least 2 years of experience working in the theatre".

**Data analysis:**

 The authors can strengthen the analysis section by providing the gender of the respondents. It is possible that the experience of the theatre nurses may differ by gender. 

**Recommendation and Conclusion:**

 The authors identified limited knowledge regarding post-exposure prophylaxis as a sub-theme that emerged from the study. This is an important finding that should be highlighted in the recommendation and conclusion section of the manuscript. Strategies to enhance the healthcare workers' knowledge regarding HIV PEP should be an important recommendation.

Is the work clearly and accurately presented and does it cite the current literature?

Partly

If applicable, is the statistical analysis and its interpretation appropriate?

Partly

Are all the source data underlying the results available to ensure full reproducibility?

Yes

Is the study design appropriate and is the work technically sound?

Yes

Are the conclusions drawn adequately supported by the results?

Partly

Are sufficient details of methods and analysis provided to allow replication by others?

Yes

Reviewer Expertise:

Sexually Transmitted and Blood Borne Infections; Social epidemiology and determinants of health; Population health intervention research; Indigenous Health; Clinical quality improvement; Epidemiology

I confirm that I have read this submission and believe that I have an appropriate level of expertise to confirm that it is of an acceptable scientific standard, however I have significant reservations, as outlined above.

# Reviewer report: Reviewer response for version 1

**DOI:** 10.5256/f1000research.137906.r191587

**Published:** 2023-09-04

**Authors:** Victoria Marcia Mutambara

**Affiliations:** 1 Livelihoods Department, Jesuit Refugee Services, Pretoria, Gauteng, South Africa

**1.** Utilizing IPA as the research design is suitable as it gives a comprehensive reflection on the participants personal perceptions.

**2.** On the discussion under the human resources theme,

***"The study also finds that not only limited nursing resources but also the anesthetist consultants are unavailable after hours. That means that the anesthetist registrar does not conduct certain functions due to difficulties in operating some equipment."***

 I would suggest the authors present their data reflecting on how these challenges are particularly specific for HIV patients as these challenges can be faced by any other patient using the hospital.

 3. " *The results did not only show the negative aspects related to infrastructure but also showed the positive aspects. The positive issues are that the blood bank is situated next to the main theatre. Though it is a bit too far from the maternity theatre, it is still a **working  distance **where an intern doctor or the general theatre worker could walk and collect blood."*

***There was a slight mistake with spelling , I assume it was supposed to be walking distance.***

 4. Whilst, the conclusion captures the main themes that emanated from the study, I would suggest the authors strengthen their conclusion and give a synthesis of the key points and also recommend areas of future research.

Is the work clearly and accurately presented and does it cite the current literature?

Yes

If applicable, is the statistical analysis and its interpretation appropriate?

Not applicable

Are all the source data underlying the results available to ensure full reproducibility?

Yes

Is the study design appropriate and is the work technically sound?

Yes

Are the conclusions drawn adequately supported by the results?

Partly

Are sufficient details of methods and analysis provided to allow replication by others?

Yes

Reviewer Expertise:

Sexual Reproductive Health, Gender and migration.

I confirm that I have read this submission and believe that I have an appropriate level of expertise to confirm that it is of an acceptable scientific standard.
